# Trends in non-suicidal self-injury among adolescents: A global cross-temporal meta-analysis, 2007–2023

**DOI:** 10.1017/S0033291725102134

**Published:** 2025-11-11

**Authors:** Jiaojiao Jia, Xiayu Du, Tieyu Duan, Zhiyu Ye, Jiawei Hu, Ting Lu, Xingyun Liu, Zongkui Zhou, Xianglian Yu, Zhihong Ren

**Affiliations:** 1 Key Laboratory of Adolescent Cyberpsychology and Behavior (CCNU), Ministry of Education, Key Laboratory of Human Development and Mental Health of Hubei Province, School of Psychology, Central China Normal University, Wuhan 430079, China; 2 Department of Psychology, Faculty of Education, Hubei University, Wuhan 430062, China; 3 Department of Education, Jianghan University, Wuhan 430056, China; 4 School of Psychology, Liaoning Normal University, Dalian 116029, China

**Keywords:** adolescent, cross-temporal meta-analysis, deliberate self-harm inventory, non-suicidal self-injury

## Abstract

Non-suicidal self-injury (NSSI) among adolescents severely jeopardizes their well-being and has emerged as a significant global public health challenge. However, research on the trends in NSSI among adolescents remains scarce. This study sought to uncover the evolving patterns in the severity of NSSI among adolescents and the factors that influence these patterns. The Deliberate Self-Harm Inventory was employed to measure the severity of NSSI among adolescents. Relevant studies were retrieved from both Chinese databases (CNKI, Wanfang, and VIP) and English databases (Web of Science, PubMed, Scopus, ProQuest, and Wiley). A total of 70 articles (71 studies; *N* = 96,382) were included in this review. The data spanned from 2007 to 2023. The analysis revealed the following: (1) Although the severity of NSSI showed a small to moderate upward trend from 2007 to 2023, this increase did not reach statistical significance. (2) No significant differences in trends were observed among Asia, Europe, and the America. (3) Adolescents with clinical samples exhibited a more pronounced upward trajectory in NSSI severity compared to those with non-clinical samples. (4) Social development indicators (GDP per capita, Human Development Index, and Internet penetration rate) and social well-being (happiness index) exhibited significant positive correlations with NSSI among adolescents. Conversely, lower social equity (higher Gini coefficient) was associated with reduced NSSI among adolescents. This study elucidated the changing trends in NSSI among adolescents and offered novel insights for the early prevention and individualized intervention of NSSI among adolescents.

## Introduction

Non-suicidal self-injury (NSSI) is defined as the deliberate and repeated infliction of direct injury to one’s own bodily tissues without suicidal intent (Klonsky, 2007). Adolescents are at a critical stage of physical and mental development (Best & Ban, 2021), which are particularly vulnerable to NSSI (Xiao et al., 2022). The study found that adolescents with NSSI, regardless of its frequency, experienced more stress and mood disorders in early adulthood compared to those without NSSI (Daukantaitė et al., 2021). NSSI is not only commonly associated with mental disorders (Hirot et al., 2025; Kaufman et al., 2024; Poudel et al., 2022; Serra et al., 2022), but it may also lead to serious consequences such as suicide (Hawton et al., 2012; Whitlock et al., 2013). In conclusion, adolescent NSSI seriously undermines their own health and has become a major challenge to global public health. Therefore, it is necessary to further investigate the development trends and influencing factors.

### Trends in NSSI over time

It is generally considered that NSSI is prevalent among adolescents (Xiao et al., 2022). A meta-analysis revealed that the global lifetime prevalence of NSSI among adolescents was 22.1% (Lim et al., 2019). The prevalence varies in different countries and regions: 27.6% in Europe (Brunner et al., 2014); 6.4%–30.8% in the United States (Monto et al., 2018); 24.7% in China (Qu et al., 2023). Despite these variations, there seems to be a consistent upward trajectory in global NSSI prevalence across recent years (Cipriano et al., 2017; Turner et al., 2022).

Longitudinal trends in NSSI severity (continuous variable) compared to NSSI prevalence (binary variable) remain inconclusive. Most findings indicate that NSSI severity tends to increase over time. For instance, a longitudinal study involving 6,092 Chinese adolescents revealed an upward trend in NSSI over two and a half years of follow-up (Xiong et al., 2023). Similar results have been reported in longitudinal studies from Canada (Daly & Willoughby, 2019), the United States (Glenn et al., 2016; Jewett et al., 2023), Spain (Faura-Garcia et al., 2024), and Australia (Scott et al., 2024). However, some follow-up studies have observed a decreasing trend in NSSI severity among high school students (Guerry & Prinstein, 2009). The discrepancies in these findings may stem from complex factors, including subject characteristics, social environments, and measurement instruments (Muehlenkamp et al., 2012). These inconsistencies suggest that NSSI may be influenced by regional distribution, a hypothesis that will be further explored in this study.

However, existing longitudinal studies typically span only a few years (Buelens et al., 2019; Marshall et al., 2013; Wei et al., 2025), which limits their ability to reflect long-term changes in NSSI severity among adolescents. Given that frequency serves as a clinical severity marker for NSSI (Ammerman et al., 2018) and can effectively predict its continuation (Brausch & Boone, 2015), further investigation into trends in NSSI frequency is warranted.

### Influencing factors of NSSI

Individual characteristics are frequently studied as NSSI influences. Longitudinal research indicates that NSSI development may fluctuate during puberty (De Luca et al., 2023). Additionally, females may exhibit higher NSSI reporting than males due to hormonal differences and gender-based variations in emotion regulation (Balzer et al., 2015; Gao et al., 2021; Nolen-Hoeksema & Aldao, 2011). Moreover, compared to non-clinical samples, adolescents with diagnosed Non-Suicidal Self-Injury Disorder (NSSID) typically display a higher frequency and greater variety of NSSI methods (Washburn et al., 2015).

Based on the modified integration model proposed by Jacobson and Batejan (2014), NSSI among adolescents involves not only individual psychological and physiological mechanisms but is also closely linked to environmental factors. However, prior studies have predominantly focused on micro-level environments, such as adverse childhood experiences and bullying (Wang et al., 2022), with limited exploration of macro-level factors. While some studies have considered social factors, their data rely predominantly on self-reports. To the best of our knowledge, no study has systematically examined the relationship between these macro-level social factors and NSSI. Therefore, this study draws on and integrates social indicators from prior researches (Miranti & Mendez, 2023; Pratama & Al-Shaikh, 2012; Rogerson, 2013; Titisari & Santoso, 2025) to analyze the macro-social factors affecting NSSI among adolescent. Specifically, GDP per capita, the Human Development Index, and Internet penetration rate are selected as proxies for social development, the Gini coefficient as a proxy for social equity, and the happiness index as a proxy for social well-being in the study.

First, social development may be inversely related to adolescent NSSI. Data from 171 countries reveal significant disparities in mental health resource allocation and accessibility across nations with varying income levels (Lora et al., 2020). This suggests that high-income countries, with their more robust psychological service systems, may better mitigate NSSI through specialized interventions such as outpatient care (Lora et al., 2020) and psychological hotlines (Matthews et al., 2023). A meta-analysis also found that NSSI prevalence is lower in developed than in developing countries (Deng et al., 2023).

However, the Internet plays a dual role in the lives of adolescents, who constitute the demographic with the highest Internet usage rates (Corcoran & Andover, 2020). While the Internet offers a platform for anonymous communication, potentially reducing social isolation (Cho, 2015), thereby possibly decreasing NSSI, a growing body of research highlights its negative impacts. Adolescents may be affected by social contagion when they are exposed to NSSI related content on the Internet (Brown & Plener, 2017). Moreover, the Internet can reinforce NSSI behaviors, provoke self-injurious impulses, and stigmatize NSSI (Brown et al., 2018).

Second, social equity may be inversely related to adolescent NSSI. According to relative deprivation theory, individuals who perceive themselves as disadvantaged may experience intense negative emotions (Smith et al., 2012) and may engage in risky behaviors (Balsa et al., 2014; Liao et al., 2024). A meta-analysis also indicated that economically disadvantaged adolescents report more mental health problems (Kim & Hagquist, 2018).

Third, social well-being may be inversely associated with adolescent NSSI. High social well-being, such as high levels of education and happiness, is positively linked to better mental health (Van Lente et al., 2012). Individuals with high well-being typically possess strong social relationships and the ability to respond adaptively to life events (Diener & Seligman, 2002), which may reduce the risk of NSSI.

### Current study

Cross-temporal meta-analysis can uncover trends in variables by integrating isolated studies chronologically (Twenge, 1997; Xin & Chi, 2008). Researchers often include studies using the same measurement tool (Karazsia et al., 2017) or different versions of the same scale such as revised or shortened versions (Buecker et al., 2021) to maintain the conceptual consistency of psychological variables across studies (Wang et al., 2024).

The Deliberate Self-Harm Inventory (DSHI), a widely used measure of non-suicidal self-injurious behaviors globally, was employed in this study (Fliege et al., 2006; Latimer et al., 2013; Vigfusdottir et al., 2022). The scale was originally developed by Gratz (2001) and comprises 17 items. Over the past two decades, various revisions and short versions of the DSHI have been created. This meta-analysis included studies utilizing the original DSHI-17 and its derivatives. These versions, while differing in length, retain the core behavioral definition and assessment approach of NSSI from the original scale, ensuring conceptual comparability. For example, the DSHI-simplified version (DSHI-s) developed by Lundh et al. (2007) improves response efficiency and is more suitable for adolescents. The DSHI-9, a subsequent revision, has also demonstrated good internal consistency and retest reliability in adolescent samples (Bjärehed & Lundh, 2008). Some researchers, based on their specific research backgrounds and previous studies (Nock, 2010; You & Lin, 2015), have excluded items less common in adolescent NSSI behaviors. By incorporating different DSHI versions, this study aims to cover diverse geographic regions, reduce version-related biases, and comprehensively explore chronological changes in adolescent NSSI (Buecker et al., 2021). The DSHI is scored by summing the item scores, with higher scores indicating greater NSSI frequency. The included studies show good internal consistency (*α* = 0.70–0.97).

This study aims to explore the trends in adolescent NSSI severity over time and the influence of individual and social factors. The following hypotheses were formulated: (1) NSSI among adolescents increases over time (Hypothesis 1). (2) Demographic variables may influence the trend of NSSI among adolescents. Specifically, female adolescents, older adolescents, and those with clinical samples may exhibit a more pronounced increase in NSSI (Hypothesis 2). (3) Social indicators may be associated with NSSI among adolescents. Specifically, higher GDP per capita, human development index, and happiness index may correlate with reduced NSSI severity, while higher Internet penetration and Gini coefficient may correlate with increased NSSI severity (Hypothesis 3). This study offers quantitative evidence on changes in adolescent NSSI severity, contributing to inform its prevention and intervention.

## Methods

This meta-analysis study is pre-registered with PROSPERO (registration number: CRD420250656170).

### Literature search

The following criteria guided literature inclusion: (a) Studies using the DSHI scale as a measurement tool. (b) Empirical studies reporting descriptive statistics (sample size, mean, and standard deviation). (c) Studies focusing on adolescents aged 10–19 (Sawyer et al., 2018). (d) For studies using follow-up data, only the first measurement was included. (e) For duplicate articles by the same author using the same dataset, only the earliest publication was selected. (f) Studies written in Chinese or English.

We searched Chinese and foreign language database such as CNKI, Wanfang, VIP, Web of Science, PubMed, Scopus, ProQuest, Wiley. Search terms included ‘adolescents’, ‘non-suicidal self-injury’, and ‘deliberate self-harm inventory’ along with their synonyms in both English and Chinese (see Supplementary Material Table A1). Literature collection concluded on December 19, 2024, yielding 70 eligible publications. These articles were published between 2008 and 2024, including 96,382 adolescent subjects. In the case of journal articles where the specific data collection years are not indicated, the data collection year (hereinafter referred to as the ‘year’) are estimated by subtracting two years from the publication year. In the case of theses, the year are estimated by subtracting one year from the actual publication year (Oliver & Hyde, 1993; Twenge, 2001; Wang et al., 2024). Our study’s data spanned from 2007 to 2023.

### Data extraction

The dataset for this study was constructed based on prior transect historical research (Wang et al., 2024) and involved the following steps. Each article was assigned a unique identifier, and basic data (including sample size, mean, and standard deviation), publication year, journal type (1 = core journal, 2 = general journal, 3 = Master’s thesis/Preprint version), year of data collection, mean age of participants, female proportion, and clinical state was coded and entered into the dataset by two psychology graduate students independently following preset coding rules. Specifically, clinical samples were defined as participants recruited from mental health institutions or diagnosed professionally, while non-clinical samples were defined as those recruited from the community or school without known mental disorders. Discrepancies were resolved through discussion.

For studies that only provided sub-study data (e.g. gender subgroups) without overall results, we used weighted synthesis of sub-study outcomes (Wang et al., 2024). Additionally, to ensure comparability of DSHI scores across versions, we performed a percent of maximum possible (POMP) transformation on the mean DSHI scores and standard deviations (Buecker et al., 2021; Cohen et al., 1999), employing these scores to assess NSSI severity.

### Quality assessment

This study employed the Joanna Briggs Institute (JBI) Critical Appraisal Checklist (Sabilillah, 2020) to systematically evaluate the quality of the included literature. Two researchers independently completed the quality coding. The checklist comprises eight assessment items, each scored as 0 or 1. The final total score ranges from 0 to 8, with higher scores indicating better study quality.

### Sources of social indicator data

The social indicators are compiled and disseminated by a variety of United Nations-affiliated organizations. The detailed sources and statistical agencies are presented in Table 1. GDP per capita serves as an indicator reflecting the economic scale and the average economic output per inhabitant of a nation or region. It is derived by dividing the gross domestic product (GDP) by the average annual population. The Human Development Index (HDI) encompasses the geometric mean of three dimensions: health, education, and income, functioning as a composite measure of a country’s overall development in these three domains. Internet penetration signifies the proportion of individuals within a population who have accessed the Internet (via fixed or mobile networks) from any location over the past three months. The Gini coefficient is an index quantifying the extent of inequality in the distribution of income or wealth, with 0.4 recognized as the international warning threshold. The Happiness Index denotes the overall satisfaction and emotional experiences of a population with their lives. It is gauged through life assessment inquiries utilizing the Cantril Self-Anchoring Striving Scale (Cantril Ladder), where individuals are requested to rate their lives on a scale of 0–10.

**Table 1.** The source of social indicator

Social indicator	Statistical agency	Source URL
GDP Per Capita	World Bank	https://data.worldbank.org.cn
Human Development Index	UNDP	https://hdr.undp.org
Internet Penetration Rate	ITU Data Hub	https://datahub.itu.int
Gini Coefficient	World Bank	https://data.worldbank.org.cn
Happiness Index	World Happiness Report	https://data.worldhappiness.report/table

### Meta-analytic procedure

Data were analyzed using R 4.4.1 (https://www.r-project.org). Initially, a linear regression model was utilized to characterize the overall trend of adolescent NSSI over time. Secondly, to better estimate the overall score, the scores were weighted by sample size (Wang et al., 2024). Then, we also statistically controlled for the effects of gender, age, clinical state, and journal type (Xin & Zhang, 2009; Wang et al., 2024). To assess the robustness of the results of this meta-analysis, this study used the Leave-One-Out (LOO) method of sensitivity analysis (Dodell-Feder & Tamir, 2018). All model fitting outcomes were visualized utilizing the ggplot2 package.

Subsequently, the change in adolescent DSHI scores was quantified based on the linear regression model results. To estimate the temporal variation in NSSI, a regression equation (Y = BX + C) was developed to predict NSSI scores, where Y represents the predicted score, B denotes the unstandardized regression coefficient, C signifies the regression intercept, and X corresponds to the year of data collection. This equation was employed to compare predicted NSSI levels between 2007 (the earliest study in the dataset) and 2023 (the most recent study). The change in NSSI was standardized by dividing it by the mean standard deviation reported in the study, with effect sizes expressed in Cohen’s *d* units. Similar cross-temporal meta-analytic procedures have been employed in prior research (e.g. Buecker et al., 2021; Curran & Hill, 2019; Twenge, 2000).

Subgroup analyses were also conducted. Given the geographical, cultural, and social normative differences across regions that may influence study outcomes (Rentfrow, 2016; Stankov, 2011), and considering that continents can serve as proxies for these regional differences (Buecker et al., 2021), this investigation adopted a continental subgroup division to examine temporal changes in adolescent NSSI across different continents.

Finally, correlation and lagged correlation analyses were performed to investigate the associations between social indicators and adolescent NSSI from three years prior, one year prior, and the current year, in that sequence(Du et al., 2025; S. Xin et al., 2023).

## Results

### Basic characteristics of the included studies

**Figure 1.**
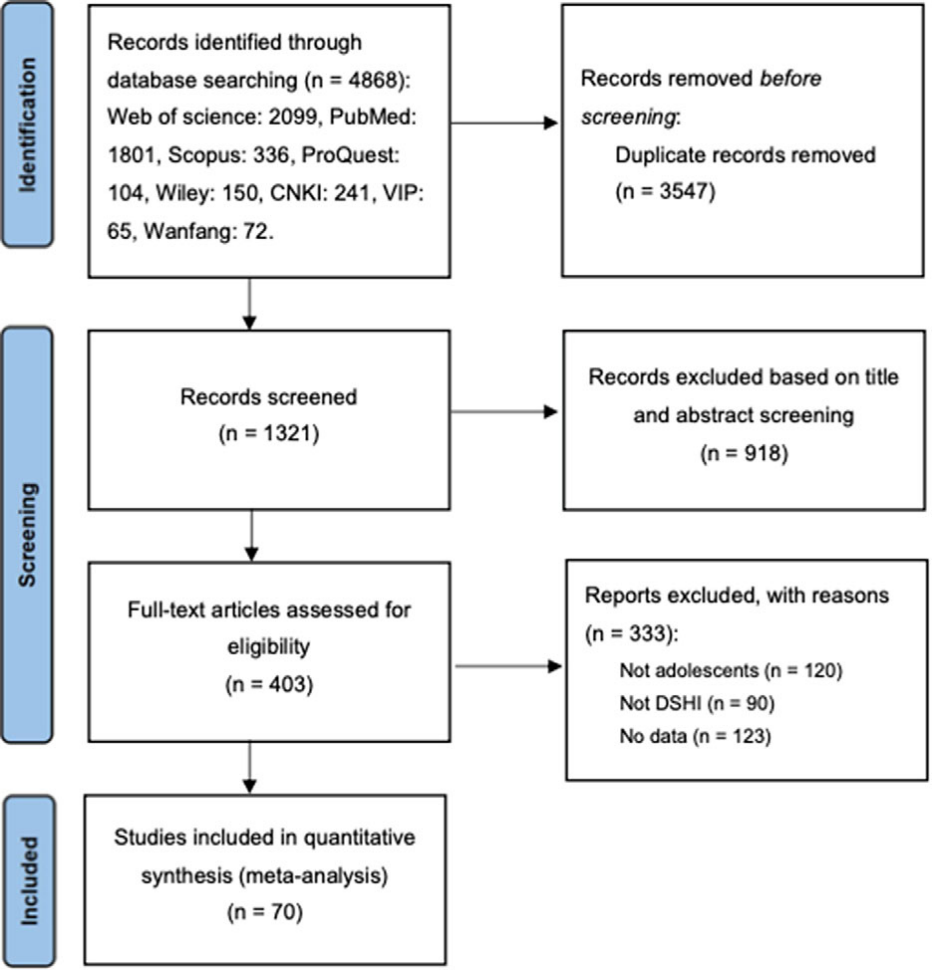
PRISMA diagram showing the results of the literature search.

The literature screening and inclusion process is presented in Figure 1, and the basic characteristics of the included studies are outlined in Table 2. Overall, 71 studies from 70 publications were incorporated, involving a total sample size of 96,382 participants. The average proportion of female participants across all independent samples was 51.14% (*SD* = 14.03), with a range spanning from 1.51% to 100%. The sample’s mean age was 14.01 years (*SD* = 1.36) and an overall range from 10.32 to 16.69 years. Clinical samples constituted approximately 11.27% of the total. The majority of the studies were conducted in Asia (*k* = 53), followed by Europe (*k* = 12), North America (*k* = 3), and Oceania (*k* = 3).

**Table 2.** Descriptions of studies included in the overall meta-analysis (71 studies in total)

Author and year	Data collection	Study location	Journal	Age mean	Female (%)	Sample size	Scale items	*M*	*SD*
Bai et al. (2024)	2017	China	1	13.47	52.00	673	9	3.08	2.50
Ojala et al. (2024)	2017	Sweden	2	15.00	93.00	166	6	35.29	77.26
Ying et al. (2024)	2020	China	1	12.34	49.30	515	12	4.86	11.85
Shen et al. (2024)	2020	China	1	12.35	51.70	453	12	4.56	10.51
Wu et al. (2024)	2020	China	1	13.4	52.70	742	11	4.12	3.21
^*^Zhang, Xie, et al. (2024)	2020	China	1	15.46	38.30	966	7	10.29	19.14
Yuan (2024)	2020	China	3	15.46	39.29	1214	7	9.91	18.75
^*^Zeng, Peng, et al. (2024)	2021	China	1	15.24	47.21	771	9	7.60	15.91
Zeng, Liu, et al. (2024)	2021	China	1	14.15	45.70	700	9	5.69	13.91
Liang et al. (2024)	2021	China	1	13.08	42.40	3290	9	2.67	2.78
Zhou et al. (2024)	2021	China	1	13.27	42.40	3098	9	2.33	2.61
Wen et al. (2024)	2022	China	1	13.41	50.65	847	12	4.17	3.08
^*^Gao, Liu, et al. (2024)	2022	China	1	13.16	53.10	838	9	8.63	15.72
Mittermeier et al. (2024)	2022	Germany	1	12.31	51.60	2117	9	0.35	1.35
Zhang, Hou, et al. (2024)	2022	China	1	12.79	42.40	646	9	4.75	4.08
Gao, Liang, et al. (2024)	2022	China	1	13.55	51.10	546	9	7.67	12.05
Yang (2024)	2022	China	1	14.62	57.40	526	7	3.52	9.05
^*^Yang & Zhao, et al. (2024)	2022	China	1	13.69	51.88	1648	7	3.05	9.52
^*^Gu et al. (2025)	2022	China	1	15.22	54.28	1319	7	4.67	3.91
Yu (2024)	2023	China	3	16.14	51.61	955	7	2.33	2.34
Zheng et al. (2023)	2018	China	1	12.32	43.87	1987	7	2.33	3.28
Xiong et al. (2023)	2019	China	1	10.32	49.00	6092	8	8.88	12.58
Hu, Peng, et al. (2023)	2020	China	2	13.71	50.38	3382	8	7.04	13.38
^*^Wang, Liu, et al. (2023)	2021	China	1	12.46	47.10	2864	11	2.00	2.53
Lin et al. (2023)	2021	China	1	15.05	40.00	1368	10	4.17	4.16
Liu et al. (2023)	2021	China	1	13.21	43.10	3561	9	23.63	16.30
^*^Hu, Zeng, et al. (2023)	2021	China	1	15.24	46.10	930	9	7.96	16.09
Larsson et al. (2023)	2021	Sweden	1	13.68	58.00	1304	9	6.83	4.22
^*^Wei et al. (2024)	2021	China	1	13.38	48.80	2381	9	4.88	3.97
Liang et al. (2023)	2021	China	1	12.36	47.10	3510	9	3.83	3.61
Bai et al. (2023)	2021	China	1	12.84	50.20	436	9	6.67	12.05
Li et al. (2023)	2021	China	1	14.38	50.95	577	7	4.67	5.17
Wang, Deng, et al. (2023)	2021	China	1	12.81	45.90	673	6	1.40	1.88
Shi (2023)	2022	China	3	15.01	50.40	711	10	1.14	4.24
Wei et al. (2023) (study 1)	2022	China	3	15.41	47.84	278	7	4.59	11.28
Wei et al. (2023) (study 2)	2022	China	3	15.4	49.04	261	7	4.00	3.53
Xiong et al. (2022)	2015	China	1	13.93	48.80	2463	9	4.75	4.00
^*^Liu, Liu, et al. (2022)	2017	China	1	12.83	52.40	564	9	4.00	7.94
Magson and Rapee (2022)	2020	Australia	1	13.8	52.00	371	12	27.61	34.16
Zhao et al. (2022)	2020	China	1	13.22	48.40	2523	11	2.20	2.65
Liu, Gao, et al. (2022)	2020	China	1	13.21	43.50	3645	9	29.17	4.44
Wang et al. (2022)	2020	China	1	12.04	1.51	5854	9	0.30	4.50
Song et al. (2022)	2020	China	1	13	44.42	1452	9	2.50	2.55
Guo et al. (2022)	2020	China	1	12.53	47.70	953	7	3.69	9.91
Morthorst et al. (2022)	2020	Denmark	2	15.03	96.70	30	6	29.02	57.39
Wei et al. (2022)	2022	China	1	15.91	52.10	643	12	2.22	5.90
Robinson et al. (2021)	2012	New Zealand	3	15.56	54.90	2057	13	0.41	0.91
Latina et al. (2021)	2014	Sweden	1	13.2	47.30	1339	9	0.22	0.81
Huang et al. (2021)	2015	China	1	12.73	42.19	859	9	4.60	0.42
Singtakaew and Chaimongkol (2021)	2019	Thailand	1	16.4194	50.60	360	10	10.17	4.72
Liu et al. (2021)	2019	China	1	12.8	52.20	536	9	4.00	2.89
Ying et al. (2021)	2019	China	1	15.92	45.30	5619	7	3.86	10.71
Wu et al. (2021)	2019	China	1	15.21	60.20	1724	7	0.50	1.48
Latina and Bayram Özdemir (2020)	2018	Sweden	1	14.42	100.00	797	9	0.33	0.72
Liu et al. (2020)	2018	China	1	13.19	46.80	2716	7	10.86	27.10
Lan et al. (2019)	2017	China	1	13.54	49.26	1210	9	3.38	2.89
Xiong et al. (2019)	2017	China	1	13.51	49.00	194	9	3.00	2.58
Wijana et al. (2018)	2012	Sweden	1	14.58	85.70	54	9	28.96	3.74
Lin et al. (2018)	2014	China	1	15.82	52.03	2170	12	1.92	6.53
Jiang et al. (2018)	2014	China	1	13.76	43.60	1026	9	1.59	4.20
Bjureberg et al. (2018)	2016	Sweden	1	15.7	76.00	25	9	18.52	17.22
Viana et al. (2018)	2016	United States	1	15.1	52.00	50	6	16.67	23.46
Somma et al. (2017)	2015	Italy	1	16.69	76.20	122	17	1.12	2.31
Thomassin et al. (2016)	2014	United States	1	14.22	58.00	95	17	2.65	54.35
Barzilay et al. (2015)	2009	Israel	1	15.91	18.90	1196	6	5.28	11.06
Garisch and Wilson (2015)	2013	New Zealand	1	16.35	43.00	1162	9	7.47	4.18
Leong et al. (2014)	2012	China	1	11.41	54.80	345	17	9.18	7.96
Marshall et al. (2013)	2011	Sweden	1	13.21	47.00	506	9	3.33	2.78
Bjärehed et al. (2012)	2007	Sweden	1	14.2	50.50	982	9	6.41	14.89
Howe-Martin et al. (2012)	2010	United States	1	16.22	50.70	211	7	1.15	1.88
Bjarehed & Lundh (2008)	2007	Sweden	1	14.1	51.09	184	19	4.43	8.28

*Note:* Journal: 1 = core journal; 2 = general journal; 3 = degree thesis. *M* = mean is converted into POMP scores. *SD* = standard deviation is converted into POMP scores.

Among them, * represents the articles included in the meta-analysis.

### Overall change in NSSI among adolescents over time

**Figure 2.**
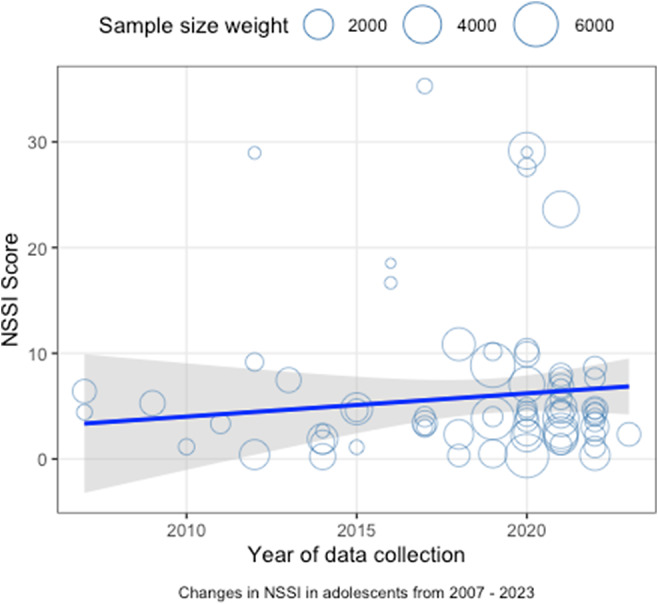
Trend of NSSI among adolescents over time.

Initially, analyses of the 71 included studies examined temporal trends in adolescent NSSI severity (Figure 2). A linear regression analysis incorporating sample size weighting revealed that year was not a significant predictor of adolescent NSSI (*b* = 0.22, *SE* = 0.26, *t* (69) = 0.41, *p* = 0.54, 95% CI = [−0.31, 0.75]). Using the statistical parameter correction method with White’s robust standard errors, the test results indicate that the negative predictive effect of age is not significant (*b* = 0.22, *SE*_Het-Robust_ = 0.21, 95% CI = [−0.20, 0.64], *R*^2^ = −0.004, *p* = 0.295). This indicates that there was no statistically significant change in the severity of NSSI among adolescents between 2007 and 2023.

Subsequently, journal type was incorporated into the linear regression model as a statistical control variable. The regression results demonstrated that journal type (*b* = −0.78, *SE* = 1.68, *t* (68) = −0.47, *p* = 0.64, 95% CI = [−4.13, 2.56]) was not a significant predictor. Moreover, year (*b* = 0.15, *SE* = 0.32, *t* (67) = 0.48, *p* = 0.63, 95% CI = [−0.56, 0.74]) continued to exert no statistically significant influence on the severity of NSSI in adolescents.

Finally, statistical controls were applied to gender, age, and clinical state. The results indicated that neither gender (*b* = −0.05, *SE* = 0.03, *t* (65) = −1.62, *p* = 0.11, 95% CI = [−0.12, 0.01]) nor age (*b* = −0.08, *SE* = 0.39, *t* (65) = −0.21, *p* = 0.83, 95% CI = [−0.87, 0.70]) significantly predicted NSSI in adolescents. In contrast, clinical state (*b* = 24.07, *SE* = 3.49, *t* (65) = 6.89, *p* < 0.001, 95% CI = [17.10, 31.05]) was a significant positive predictor. At this stage, year (*b* = 0.12, *SE* = 0.13, *t* (65) = 0.91, *p* = 0.37, 95% CI = [−0.14, 0.38]) remained a non-significant positive factor. To conclude, these results suggest that the severity of NSSI among adolescents has not changed significantly from 2007 to 2023.

The sensitivity analysis revealed that excluding individual samples had minimal impact on the results. The *b*-coefficients and *p*-values of the model showed no significant changes, indicating the model’s robustness (see Supplementary Material Figure A1). Random-effects pooling revealed substantial between-study heterogeneity (*Q* (5) =382.46, *p* < .001; *I*^2^ = 98.9%).

### Amount of change in NSSI among adolescents over time

Based on the linear regression model results, the effect size *d* was expressed as a standardized score (Buecker et al., 2021). The linear regression analysis yielded an approximate equation of *Y* = 0.22X − 437.23. Subsequently, the *Z*-scores for adolescent NSSI were calculated by substituting the years 2007 and 2023 into the regression equation, resulting in values of 4.31 and 7.83 respectively. Finally, the difference between *Z_2023_* and *Z_2007_* was computed and divided by the mean standard deviation of the *Z*-scores over the 16-year period, which was 9.99, to obtain a d-value of 0.35. These findings indicate that the severity of NSSI among adolescents increased by 0.35 standardized scores from 2007 to 2023. According to established criteria where ‘0.2 represents a small effect size, 0.5 a medium effect size, and 0.8 a large effect size’ (Buecker et al., 2021; Cohen, 1977; Twenge, 2001), the increase in adolescent NSSI severity from 2007 to 2023 corresponds to a small to medium effect size.

### Subgroup analysis

Subgroup analyses were conducted to examine potential continental differences in temporal trends of NSSI. The results indicated that relative to European samples, American samples exhibited a non-significant effect size difference of −6.52 (95% CI [−44.67, 31.62]), Asian samples showed a non-significant difference of 4.14 (95% CI [−3.39, 11.66]), and Oceanian samples demonstrated a non-significant difference of 5.94 (95% CI [−9.11, 20.98]). White’s robust standard error test shows that the age effect remains insignificant (*b* = 0.14, *SE*_Het-Robust_ = 0.24, 95% CI = [−0.34, 0.61], *p* = 0.572). Therefore, NSSI changes with year did not significantly differ across continents. These continental comparisons were exploratory in nature, so no specific hypotheses were predefined.

### Correlations between adolescent NSSI and Social Indicators

The correlation and lagged correlation analyses revealed that social development indicators (GDP per capita, HDI, and Internet penetration), social equity (Gini coefficient), and social well-being (Happiness Index) exhibited significant correlations with adolescent NSSI (see Table 3).

**Table 3.** Correlations between social indicators and adolescent NSSI

Social indicator	3 years prior		1 year prior		Actual year	
	N	*r*	N	*r*	N	*r*
GDP	70	0.397**	70	0.409**	70	0.397**
HDI	70	0.365**	70	0.366**	69	0.368**
Gini	66	−0.390**	65	−0.356**	54	−0.391**
INT	70	0.384**	70	0.370**	70	0.414**
HAP	59	0.428**	61	0.526**	65	0.447**

*Note*: *p < 0.05, **p < 0.01; GDP = GDP Per Capita; HDI = Human Development Index; INT = Internet Penetration Rate; Gini = Gini Coefficient; HAP = Happiness Index

### Quality assessment

The Spearman correlation coefficient between the two raters was 0.75 (*p* < 0.01), indicating a strong level of agreement. The quality assessment revealed that the included studies had a mean score of 6.55 ± 0.86, with individual scores ranging from 5 to 8, suggesting that all studies were of moderate to high quality. The specific item scores after resolving the disagreements are presented in Supplementary Material Table A2. Subsequent analyses revealed that the moderating effects of the quality assessment score were not statistically significant (*b* = −0.05, *SE* = 0.34, *t* (67) = −0.16, *p* = 0.88, 95% CI = [−0.72, 0.62]). Therefore, to maintain the integrity of the evidence, no studies were excluded based on quality scores.

## Discussion

This study employed a cross-temporal meta-analysis to examine trends in adolescent NSSI and the influencing factors from 2007 to 2023. The results revealed that the severity of NSSI exhibited a small to moderate upward trend from 2007 to 2023, but this trend was not statistically significant. Subgroup analyses indicated no significant continental variation in NSSI trends. Furthermore, gender, social development, social equity, and social well-being significantly impacted these trends. These findings enhance our understanding of adolescent NSSI and may inform effective intervention strategies.

### Change in of NSSI among adolescents over time

This study found that the severity of NSSI among adolescents exhibited a non-significant change from 2007 to 2023, contradicting Hypothesis 1. This may be due to the offsetting effect between the risk factors and protective factors for NSSI. Adolescents face global risk factors like COVID-19, climate crises, and scarce mental health resources (Lass-Hennemann et al., 2024), as well as individual-specific factors such as parent–child and peer relationships (Brown & Witt, 2019). These factors may elevate the severity of NSSI in adolescents.

However, in recent years, there has been increasing global attention on adolescent mental health, with rising expenditures on related services (Brazel et al., 2023; Fullman et al., 2018), a trend that is beneficial for improving the mental health of adolescents (Zaneva et al., 2022). Adolescents can access mental health services through multiple channels, including schools, hospital, and juvenile justice agencies (Duong et al., 2021). Countries have also established 24-hour public psychological hotlines or online chat platforms, such as the 988 Suicide & Crisis Lifeline in the United States, New Zealand’s 1737 Need to Talk, and Sweden’s Mind. Notably, during the COVID-19, the workload and utilization of mental crisis hotlines increased significantly, partially meeting mental health needs (Arendt et al., 2020; Zhou et al., 2020). All these factors collectively buffer the adolescent NSSI process.

### Differences in NSSI among adolescents over time by continent

When examining temporal trends in adolescent NSSI severity, no significant differences were observed across continents, which may suggest that adolescent NSSI constitutes a serious global public health problem. However, this does not imply that other characteristics of adolescent NSSI are uniform across continents. Previous cross-cultural studies have demonstrated that method of NSSI exhibits cultural variation (Gholamrezaei et al., 2017). For instance, compared with Belgium, Indian samples reported more head banging and less cutting and scratching (Gandhi et al., 2021). Furthermore, the relatively low number of studies from North America and Oceania may influence the effect size of the present findings and warrants caution when generalizing these results.

### Individual characteristics influencing NSSI changes among adolescents

This study indicates that clinical samples adolescents exhibit higher levels of self-injury severity compared to non-clinical samples. This heightened NSSI severity may be related to the co-occurring psychiatric disorders and greater symptom severity of clinical samples. In the included studies, clinical samples frequently presented with one or more additional psychiatric disorders (Morthorst et al., 2022; Ojala et al., 2024; Viana et al., 2018).

The results indicated no significant gender effect on adolescent NSSI, contradicting Hypothesis 2. However, this finding aligns with previous research (Gratz et al., 2012; Taliaferro et al., 2023; Zhao et al., 2024). While consensus remains elusive regarding gender’s impact on NSSI frequency and incidence, more uniform evidence suggests gender influences NSSI methods (Andrei et al., 2024; Calvete et al., 2015; Lundh et al., 2007; Moloney et al., 2025). Similarly, age showed no significant association with adolescent NSSI. This may suggest a possible complex nonlinear relationship between age and NSSI. Wilkinson et al. (2022) found a complex interaction among age, gender, and NSSI, with general distress serving as a partial mediator.

### Relationship of social Indicators to NSSI among adolescents

This study examined the associations between three types of societal indicators and adolescent NSSI, thereby expanding understanding of its social etiology. This study revealed that higher levels of social development are associated with increased likelihood of NSSI behaviors among adolescents, a finding that contradicts the hypothesis. Two complementary mechanisms may account for this paradox. Specifically, in societies with advanced economic and overall development, individuals typically undergo more years of schooling. This extended education exposes adolescents to heightened academic stress Sathish & Subramanian, 2021. The fast-paced lifestyle and overwhelming academic workload may lead to sleep deprivation in adolescents, further worsening their emotion regulation capabilities (Mazza et al., 2024). The widespread use of the Internet exacerbates this risk, a result consistent with prior studies (Mészáros et al., 2022; Yang et al., 2020). When using social media, adolescents may encounter NSSI - related content, which may trigger imitation. Moreover, within certain online subcultural communities, NSSI can be symbolized as a marker of identity, potentially prompting adolescents to engage in such behaviors to seek peer recognition and group belonging (Brown et al., 2020). On the other hand, these regions typically establish stronger mental-health infrastructure, train more school counsellors, provide more accessible psychological service resources (Ndetei et al., 2023; Wolthusen & Andrä, 2023). Consequently, it is both contributed to enhanced early identification and reporting capacity for NSSI.

Interestingly, lower social equity (reflected by a higher Gini coefficient) was associated with reduced adolescent NSSI, contradicting the hypothesis. As Adams’ (1963) equity theory, macro-level equity does not inherently ensure micro-level equity. That is to say, even when the Gini coefficient is low, adolescents may still experience a sense of relative deprivation due to micro-level differences such as parental companionship, and adverse childhood experiences (Tian et al., 2023; Wang & Meng, 2024), which in turn increases the risk of NSSI.

Similarly, social well-being presents a positive correlation with adolescent NSSI, conflicting with the hypothesis. Unlike prior research that predominantly examined happiness at the individual level (Diener & Seligman, 2002; Van Lente et al., 2012), the present study utilized a national-level happiness index. This macro-level indicator may capture broader societal conditions, which could help explain the counterintuitive association. From the perspective of mental health destigmatization, countries with elevated well-being indices may prioritize mental health, fostering social openness and acceptance of psychological issues. Adolescents in these countries might encounter reduced mental health stigma (World Happiness Report, n.d.), prompting more honest self-reports of negative emotions and enhancing help-seeking behaviors (Crockett et al., 2025). Moreover, this situation could also stem from statistical bias.

### Significance and limitations

This study offers significant implications for comprehending and addressing adolescent NSSI. First, this study pioneeringly applied cross-temporal meta-analysis to reveal the temporal trends in NSSI from the perspective of frequency. Unlike previous studies that only focused on NSSI prevalence as a categorical variable, the cross-temporal meta-analysis of NSSI frequency as a continuous variable can more finely reveal its changing trends, deepening the understanding of adolescent NSSI. Secondly, the study examined not only individual characteristics but also emphasized the relationship between macro - social indicators and NSSI. This offers a new perspective on understanding adolescent NSSI and provides a scientific basis for devising targeted social policies and intervention measures to prevent and reduce such behaviors in adolescents. Specifically, it suggests developing differentiated intervention programs tailored to different regions and social factors, and providing more intensive interventions for the clinical samples.

Although enhancing our comprehension of adolescent NSSI trends, this study has certain limitations. Firstly, only literature utilizing the DSHI scale was analyzed. Future research could incorporate other tools like the ISAS, OSI, and FASM for comparison. Secondly, the study focused solely on NSSI frequency trends, leaving other characteristics unexplored. Future research could examine changes in NSSI methods and the emerging phenomenon of digital self-harm (Patchin et al., 2023). Third, the language inclusion criteria (Chinese and English only) and the geographic origins of the samples may limit the generalizability of our results. Specifically, the Asian population was overrepresented, while data from Europe, the Americas, and Oceania were limited. Studies from Africa were absent. Future studies should explore cross-cultural differences through multinational, multicenter follow-up research to clarify how sociocultural factors impact adolescent NSSI.

## General Conclusions

This cross-temporal meta-analysis examined trends in adolescent NSSI and its influencing factors. The results indicated: (1) No significant change occurred in the severity of adolescent NSSI from 2007 to 2023 (*d* = 0.35). (2) No significant differences were found in NSSI trends across Asia, Europe, and the Americas. (3) NSSI severity changes were more pronounced in clinical samples than in non-clinical samples. (4) Social indicators showed a close relationship with adolescent NSSI. Specifically, social development (GDP per capita, HDI, and Internet penetration) and social well-being (happiness index) were significantly positively correlated with adolescent NSSI, whereas lower social equity (higher Gini coefficient) was associated with reduced adolescent NSSI.

## Supporting information

Jia et al. supplementary material Jia et al. supplementary material

## Supplementary material

The supplementary material for this article can be http://doi.org/10.1017/S0033291725102134.
