# Supplementary material for: Trends in non-suicidal self-injury among adolescents: A global cross-temporal meta-analysis, 2007–2023
Source: Psychol Med. 2025 Nov 11;55:e340. doi: 10.1017/S0033291725102134 (PMC13058657; doi:10.1017/S0033291725102134)
Supplement: Jia et al. supplementary material [file S0033291725102134sup001.docx]

**Trends in Non-Suicidal Self-Injury (NSSI) among Adolescents: A Global Cross-Temporal Meta-Analysis, 2007–2023**

**Online Supplementary Material**

**Table A1.** Search Strategies in Different Databases

**Table A2.** Study quality assessment scores (71 studies in total)

**Table A3.** PRISMA checklist

**Figure. A1.** Sensitivity Analysis of Linear Regression Model

**Table A1.** Search Strategies in Different Databases

| Database | Search strategy |
| --- | --- |
| CNKI | (AB=('青少年'+'青春期'+'学校'+'未成年人'+'初中生'+'高中生'+'中学生') and AB=('非自杀性自伤'+'自残'+'自我伤害')) and FT=('自伤行为量表'+'蓄意自伤量表'+'自伤行为问卷'+'蓄意自伤问卷'+'Deliberate Self‐Harm Inventory'+'DSHI') |
| Wanfang | (主题:("青少年" OR "未成年人" OR "初中生" OR "高中生" OR "中学生") and 主题:(\“非自杀性自伤\” OR "自残" OR "自我伤害") and 全部:(\“自伤行为量表\” OR \“自伤行为问卷\” OR \“蓄意自伤量表\” OR \“蓄意自伤问卷\” OR \“deliberate self‐harm inventory\” OR \“DSHI\” OR \“故意自伤量表\” OR \“故意自伤问卷\”)) |
| VIP | (R="青少年" or R="青春期" or R="学校" or R="未成年人" or R="初中生" or R="高中生" or R="中学生") and (R="非自杀性自伤" or R="自残" or R="自我伤害") and (R="自伤行为量表" or R="蓄意自伤量表" or R="自伤行为问卷" or R="蓄意自伤问卷" or R="故意自伤量表" or R="故意自伤问卷" or R="deliberate self‐harm inventory" or R="DSHI") |
| Scopus | (TITLE-ABS-KEY("non-suicidal self-injur*") OR TITLE-ABS-KEY("non-suicidal self injur*") OR TITLE-ABS-KEY("nonsuicidal self injur*") OR TITLE-ABS-KEY("non suicidal self injury") OR TITLE-ABS-KEY("NSSI") OR TITLE-ABS-KEY("self harm") OR TITLE-ABS-KEY("self-harm") OR TITLE-ABS-KEY("self-injur*") OR TITLE-ABS-KEY("self injur*") OR TITLE-ABS-KEY("self-destructive behavio*") OR TITLE-ABS-KEY("self-destructive") OR TITLE-ABS-KEY("self destructive behavio*") OR TITLE-ABS-KEY("self destructive behavior") AND (TITLE-ABS-KEY("adolescen*") OR TITLE-ABS-KEY("youth*") OR TITLE-ABS-KEY("teen*) AND (ALL("deliberate self-harm inventory") OR ALL("DSHI")) |
| Web of science | AB=((adolescen* OR teen* OR youth*)) AND AB=(("non-suicidal self-injur*" OR "non-suicidal self injur*" OR "nonsuicidal self injur*" OR "non suicidal self injury" OR "NSSI" OR "self harm" OR "self-harm" OR "self-injur*" OR "self injur*" OR "self-destructive behavio*" OR " self-destructive" OR "self destructive behavio*" OR "self destructive Behavior" OR "deliberate self harm" OR "deliberate self-harm")) |
| PubMed | ("adolescen*"[Title/Abstract] OR "teen*"[Title/Abstract] OR "youth*"[Title/Abstract]) AND ("non suicidal self injur*"[Title/Abstract] OR "non suicidal self injur*"[Title/Abstract] OR "nonsuicidal self injur*"[Title/Abstract] OR "non suicidal self injury"[Title/Abstract] OR "NSSI"[Title/Abstract] OR "self harm"[Title/Abstract] OR "self harm"[Title/Abstract] OR "self injur*"[Title/Abstract] OR "self injur*"[Title/Abstract] OR "self destructive behavio*"[Title/Abstract] OR "self-destructive"[Title/Abstract] OR "self destructive behavio*"[Title/Abstract] OR "self destructive behavior"[Title/Abstract] OR "deliberate self harm"[Title/Abstract] OR "deliberate self harm"[Title/Abstract]) |
| Wiley | "adolescen*" OR "teen*" OR "youth*"" in Abstract and "non-suicidal self-injur*" OR "non-suicidal self injur*" OR "nonsuicidal self injur*" OR "non suicidal self injury" OR "NSSI" OR "self harm" OR "self-harm" OR "self-injur*" OR "self injur*" OR "self-destructive behavio*" OR "self-destructive" OR "self destructive behavio*" OR "self destructive behavior" OR "deliberate self harm" OR "deliberate self-harm" in Abstract |
| ProQuest | summary("adolescen*" OR "teen*" OR "youth*") AND summary("non-suicidal self-injur*" OR "non-suicidal self injur*" OR "nonsuicidal self injur*" OR "non suicidal self injury" OR "NSSI" OR "self harm" OR "self-harm" OR "self-injur*" OR "self injur*" OR "self-destructive behavio*" OR " self-destructive" OR "self destructive behavio*" OR "self destructive behavior" OR "deliberate self harm" OR "deliberate self-harm") AND ("deliberate self-harm inventory" OR "DSHI") |

**Table A2.** Study quality assessment scores (71 studies in total)

| Authors and Year | Item 1 | Item 2 | Item 3 | Item 4 | Item 5 | Item 6 | Item 7 | Item 8 | In total |
| --- | --- | --- | --- | --- | --- | --- | --- | --- | --- |
| Bai et al. (2024) | 0 | 1 | 1 | 1 | 1 | 1 | 1 | 1 | 7 |
| Gao, liang et al. (2024) | 0 | 1 | 1 | 1 | 1 | 1 | 1 | 1 | 7 |
| Gao, Liu et al. (2024) | 1 | 1 | 1 | 1 | 1 | 1 | 1 | 1 | 8 |
| Gu et al. (2024) | 0 | 1 | 1 | 1 | 0 | 0 | 1 | 1 | 5 |
| Liang et al. (2024) | 0 | 1 | 1 | 1 | 1 | 1 | 1 | 1 | 7 |
| Mittermeier et al. (2024) | 1 | 1 | 1 | 1 | 1 | 1 | 1 | 1 | 8 |
| Ojala et al. (2024) | 1 | 0 | 0 | 1 | 1 | 1 | 1 | 1 | 6 |
| Shen et al. (2024) | 0 | 1 | 1 | 1 | 1 | 1 | 1 | 1 | 7 |
| Wen et al. (2024) | 0 | 1 | 1 | 1 | 1 | 1 | 1 | 1 | 7 |
| Wu et al. (2024) | 0 | 1 | 1 | 1 | 1 | 0 | 1 | 1 | 6 |
| Yang & Zhao (2024) | 0 | 1 | 1 | 1 | 1 | 1 | 1 | 1 | 7 |
| Yang (2024) | 0 | 1 | 1 | 1 | 1 | 1 | 1 | 1 | 7 |
| Ying et al. (2024) | 0 | 1 | 1 | 1 | 1 | 1 | 1 | 1 | 7 |
| Yu (2024) | 0 | 1 | 1 | 1 | 1 | 0 | 1 | 1 | 6 |
| Yuan (2024) | 0 | 1 | 1 | 1 | 1 | 1 | 1 | 1 | 7 |
| Zeng, Liu et al. (2024) | 0 | 1 | 1 | 1 | 1 | 0 | 1 | 1 | 6 |
| Zeng, Peng et al. (2024) | 1 | 1 | 1 | 1 | 1 | 1 | 1 | 1 | 8 |
| Zhang, Hou et al. (2024) | 0 | 1 | 1 | 1 | 1 | 1 | 1 | 1 | 7 |
| Zhang, Xie et al. (2024) | 0 | 1 | 1 | 1 | 1 | 1 | 1 | 1 | 7 |
| Zhou et al. (2024) | 0 | 1 | 1 | 1 | 1 | 1 | 1 | 1 | 7 |
| Bai et al. (2023) | 0 | 1 | 0 | 1 | 1 | 0 | 1 | 1 | 5 |
| Hu, Peng et al. (2023) | 0 | 0 | 1 | 1 | 1 | 1 | 1 | 1 | 6 |
| Hu, Zeng et al. (2023) | 0 | 1 | 1 | 1 | 1 | 1 | 1 | 1 | 7 |
| Larsson et al. (2023) | 1 | 1 | 1 | 1 | 1 | 1 | 1 | 1 | 8 |
| Li et al. (2023) | 0 | 1 | 1 | 1 | 1 | 1 | 1 | 1 | 7 |
| Liang et al. (2023) | 0 | 1 | 1 | 1 | 1 | 1 | 1 | 1 | 7 |
| Lin et al. (2023) | 0 | 0 | 1 | 1 | 1 | 1 | 1 | 1 | 6 |
| Liu et al. (2023) | 0 | 1 | 1 | 1 | 1 | 1 | 1 | 1 | 7 |
| Shi (2023) | 0 | 1 | 1 | 1 | 1 | 1 | 1 | 1 | 7 |
| Wang, Deng et al. (2023) | 0 | 0 | 1 | 1 | 1 | 0 | 1 | 1 | 5 |
| Wang, Liu et al. (2023) | 0 | 1 | 1 | 1 | 1 | 1 | 1 | 1 | 7 |
| Wei (2023) (study 1) | 0 | 1 | 1 | 1 | 1 | 1 | 1 | 1 | 7 |
| Wei (2023) (study2) | 0 | 1 | 1 | 1 | 0 | 0 | 1 | 1 | 5 |
| Wei et al. (2023) | 0 | 1 | 1 | 1 | 1 | 0 | 1 | 1 | 6 |
| Xiong et al. (2023) | 0 | 1 | 1 | 1 | 1 | 1 | 1 | 1 | 7 |
| Zheng et al. (2023) | 0 | 0 | 1 | 1 | 1 | 0 | 1 | 1 | 5 |
| Guo et al. (2022) | 0 | 0 | 1 | 1 | 1 | 0 | 1 | 1 | 5 |
| Liu, Gao et al. (2022) | 0 | 1 | 1 | 1 | 1 | 1 | 1 | 1 | 7 |
| Liu, Liu et al. (2022) | 0 | 1 | 1 | 1 | 1 | 1 | 1 | 1 | 7 |
| Magson & Rapee (2022) | 1 | 0 | 1 | 1 | 1 | 1 | 1 | 1 | 7 |
| Morthorst et al. (2022) | 1 | 0 | 0 | 1 | 1 | 1 | 1 | 1 | 6 |
| Song et al. (2022) | 0 | 1 | 1 | 1 | 1 | 0 | 1 | 1 | 6 |
| Wang et al. (2022) | 0 | 0 | 1 | 1 | 1 | 0 | 1 | 1 | 5 |
| Wei et al. (2022) | 0 | 1 | 1 | 1 | 1 | 0 | 1 | 1 | 6 |
| Xiong et al. (2022) | 0 | 1 | 1 | 1 | 1 | 1 | 1 | 1 | 7 |
| Zhao et al. (2022) | 0 | 0 | 1 | 1 | 1 | 1 | 1 | 1 | 6 |
| Huang et al. (2022) | 0 | 1 | 1 | 1 | 1 | 0 | 1 | 1 | 6 |
| Latina et al. (2022) | 0 | 1 | 1 | 1 | 1 | 0 | 1 | 1 | 6 |
| Liu et al. (2022) | 0 | 1 | 1 | 1 | 1 | 1 | 1 | 1 | 7 |
| Robinson et al. (2021) | 0 | 1 | 1 | 1 | 1 | 0 | 1 | 1 | 6 |
| Singtakaew & Chaimongkol (2021) | 1 | 1 | 1 | 1 | 1 | 1 | 1 | 1 | 8 |
| Wu et al. (2021) | 0 | 1 | 1 | 1 | 1 | 0 | 1 | 1 | 6 |
| Ying et al. (2021) | 0 | 0 | 1 | 1 | 1 | 1 | 1 | 1 | 6 |
| Latina et al. (2021) | 0 | 1 | 1 | 1 | 1 | 1 | 1 | 1 | 7 |
| Liu, You et al. (2021) | 0 | 1 | 1 | 1 | 1 | 0 | 1 | 1 | 6 |
| Lan et al. (2021) | 1 | 1 | 1 | 1 | 1 | 0 | 1 | 1 | 7 |
| Xiong et al. (2021) | 1 | 1 | 1 | 1 | 1 | 1 | 1 | 1 | 8 |
| Bjureberg et al. (2018) | 1 | 1 | 1 | 1 | 1 | 1 | 1 | 1 | 8 |
| Jiang et al. (2018) | 0 | 1 | 1 | 1 | 1 | 1 | 1 | 1 | 7 |
| Lin et al. (2018) | 0 | 1 | 1 | 1 | 1 | 0 | 1 | 1 | 6 |
| Viana et al. (2018) | 1 | 0 | 1 | 1 | 1 | 0 | 1 | 1 | 6 |
| Wijana et al. (2018) | 1 | 1 | 1 | 1 | 1 | 1 | 1 | 1 | 8 |
| Somma et al. (2017) | 0 | 0 | 1 | 1 | 1 | 0 | 1 | 1 | 5 |
| Thomassin et al. (2016) | 1 | 0 | 1 | 1 | 1 | 1 | 1 | 1 | 7 |
| Barzilay et al. (2016) | 0 | 0 | 0 | 1 | 1 | 1 | 1 | 1 | 5 |
| Garisch & Wilson (2015) | 0 | 1 | 0 | 1 | 1 | 1 | 1 | 1 | 6 |
| Leong et al. (2014) | 0 | 1 | 1 | 1 | 1 | 1 | 1 | 1 | 7 |
| Marshall et al. (2013) | 0 | 1 | 1 | 1 | 1 | 0 | 1 | 1 | 6 |
| Bjärehed et al. (2012) | 0 | 1 | 1 | 1 | 1 | 1 | 1 | 1 | 7 |
| Howe-Martin et al. (2012) | 0 | 1 | 1 | 1 | 1 | 0 | 1 | 1 | 6 |
| Bjarehed & Lundh (2008) | 0 | 1 | 1 | 1 | 1 | 1 | 1 | 1 | 7 |

**Table A3.** PRISMA checklist

| **Section and Topic** | **Item #** | **Checklist item** | **Location where item is reported** |
| --- | --- | --- | --- |
| **TITLE** | | |  |
| Title | 1 | Identify the report as a systematic review. | p1 |
| **ABSTRACT** | | |  |
| Abstract | 2 | See the PRISMA 2020 for Abstracts checklist. | p2 |
| **INTRODUCTION** | | |  |
| Rationale | 3 | Describe the rationale for the review in the context of existing knowledge. | p3-6 |
| Objectives | 4 | Provide an explicit statement of the objective(s) or question(s) the review addresses. | p6-7 |
| **METHODS** | | |  |
| Eligibility criteria | 5 | Specify the inclusion and exclusion criteria for the review and how studies were grouped for the syntheses. | p9 |
| Information sources | 6 | Specify all databases, registers, websites, organisations, reference lists and other sources searched or consulted to identify studies. Specify the date when each source was last searched or consulted. | p10 |
| Search strategy | 7 | Present the full search strategies for all databases, registers and websites, including any filters and limits used. | p10 |
| Selection process | 8 | Specify the methods used to decide whether a study met the inclusion criteria of the review, including how many reviewers screened each record and each report retrieved, whether they worked independently, and if applicable, details of automation tools used in the process. | p10-11 |
| Data collection process | 9 | Specify the methods used to collect data from reports, including how many reviewers collected data from each report, whether they worked independently, any processes for obtaining or confirming data from study investigators, and if applicable, details of automation tools used in the process. | p11 |
| Data items | 10a | List and define all outcomes for which data were sought. Specify whether all results that were compatible with each outcome domain in each study were sought (e.g. for all measures, time points, analyses), and if not, the methods used to decide which results to collect. | p11 |
|  | 10b | List and define all other variables for which data were sought (e.g. participant and intervention characteristics, funding sources). Describe any assumptions made about any missing or unclear information. | p11 |
| Study risk of bias assessment | 11 | Specify the methods used to assess risk of bias in the included studies, including details of the tool(s) used, how many reviewers assessed each study and whether they worked independently, and if applicable, details of automation tools used in the process. | p11 |
| Effect measures | 12 | Specify for each outcome the effect measure(s) (e.g. risk ratio, mean difference) used in the synthesis or presentation of results. | p12-13 |
| Synthesis methods | 13a | Describe the processes used to decide which studies were eligible for each synthesis (e.g. tabulating the study intervention characteristics and comparing against the planned groups for each synthesis (item #5)). | p12-13 |
|  | 13b | Describe any methods required to prepare the data for presentation or synthesis, such as handling of missing summary statistics, or data conversions. | p10 |
|  | 13c | Describe any methods used to tabulate or visually display results of individual studies and syntheses. | p12-13 |
|  | 13d | Describe any methods used to synthesize results and provide a rationale for the choice(s). If meta-analysis was performed, describe the model(s), method(s) to identify the presence and extent of statistical heterogeneity, and software package(s) used. | p12-13 |
|  | 13e | Describe any methods used to explore possible causes of heterogeneity among study results (e.g. subgroup analysis, meta-regression). | p12-13 |
|  | 13f | Describe any sensitivity analyses conducted to assess robustness of the synthesized results. | p12-13 |
| Reporting bias assessment | 14 | Describe any methods used to assess risk of bias due to missing results in a synthesis (arising from reporting biases). | p10-11 |
| Certainty assessment | 15 | Describe any methods used to assess certainty (or confidence) in the body of evidence for an outcome. | p12-13 |
| **RESULTS** | | |  |
| Study selection | 16a | Describe the results of the search and selection process, from the number of records identified in the search to the number of studies included in the review, ideally using a flow diagram. | p14-15, 59 |
|  | 16b | Cite studies that might appear to meet the inclusion criteria, but which were excluded, and explain why they were excluded. | No |
| Study characteristics | 17 | Cite each included study and present its characteristics. | P56-58 |
| Risk of bias in studies | 18 | Present assessments of risk of bias for each included study. | p16 |
| Results of individual studies | 19 | For all outcomes, present, for each study: (a) summary statistics for each group (where appropriate) and (b) an effect estimate and its precision (e.g. confidence/credible interval), ideally using structured tables or plots. | P56-58 |
| Results of syntheses | 20a | For each synthesis, briefly summarise the characteristics and risk of bias among contributing studies. | P56-58 |
|  | 20b | Present results of all statistical syntheses conducted. If meta-analysis was done, present for each the summary estimate and its precision (e.g. confidence/credible interval) and measures of statistical heterogeneity. If comparing groups, describe the direction of the effect. | p14-16 |
|  | 20c | Present results of all investigations of possible causes of heterogeneity among study results. | p13-15 |
|  | 20d | Present results of all sensitivity analyses conducted to assess the robustness of the synthesized results. | No |
| Reporting biases | 21 | Present assessments of risk of bias due to missing results (arising from reporting biases) for each synthesis assessed. | p15 |
| Certainty of evidence | 22 | Present assessments of certainty (or confidence) in the body of evidence for each outcome assessed. | p15 |
| **DISCUSSION** | | |  |
| Discussion | 23a | Provide a general interpretation of the results in the context of other evidence. | p17-20 |
|  | 23b | Discuss any limitations of the evidence included in the review. | P21-22 |
|  | 23c | Discuss any limitations of the review processes used. | P21-22 |
|  | 23d | Discuss implications of the results for practice, policy, and future research. | P21 |
| **OTHER INFORMATION** | | |  |
| Registration and protocol | 24a | Provide registration information for the review, including register name and registration number, or state that the review was not registered. | p8 |
|  | 24b | Indicate where the review protocol can be accessed, or state that a protocol was not prepared. | p8 |
|  | 24c | Describe and explain any amendments to information provided at registration or in the protocol. | p8 |
| Support | 25 | Describe sources of financial or non-financial support for the review, and the role of the funders or sponsors in the review. | p23 |
| Competing interests | 26 | Declare any competing interests of review authors. | p23 |
| Availability of data, code and other materials | 27 | Report which of the following are publicly available and where they can be found: template data collection forms; data extracted from included studies; data used for all analyses; analytic code; any other materials used in the review. | p23 |


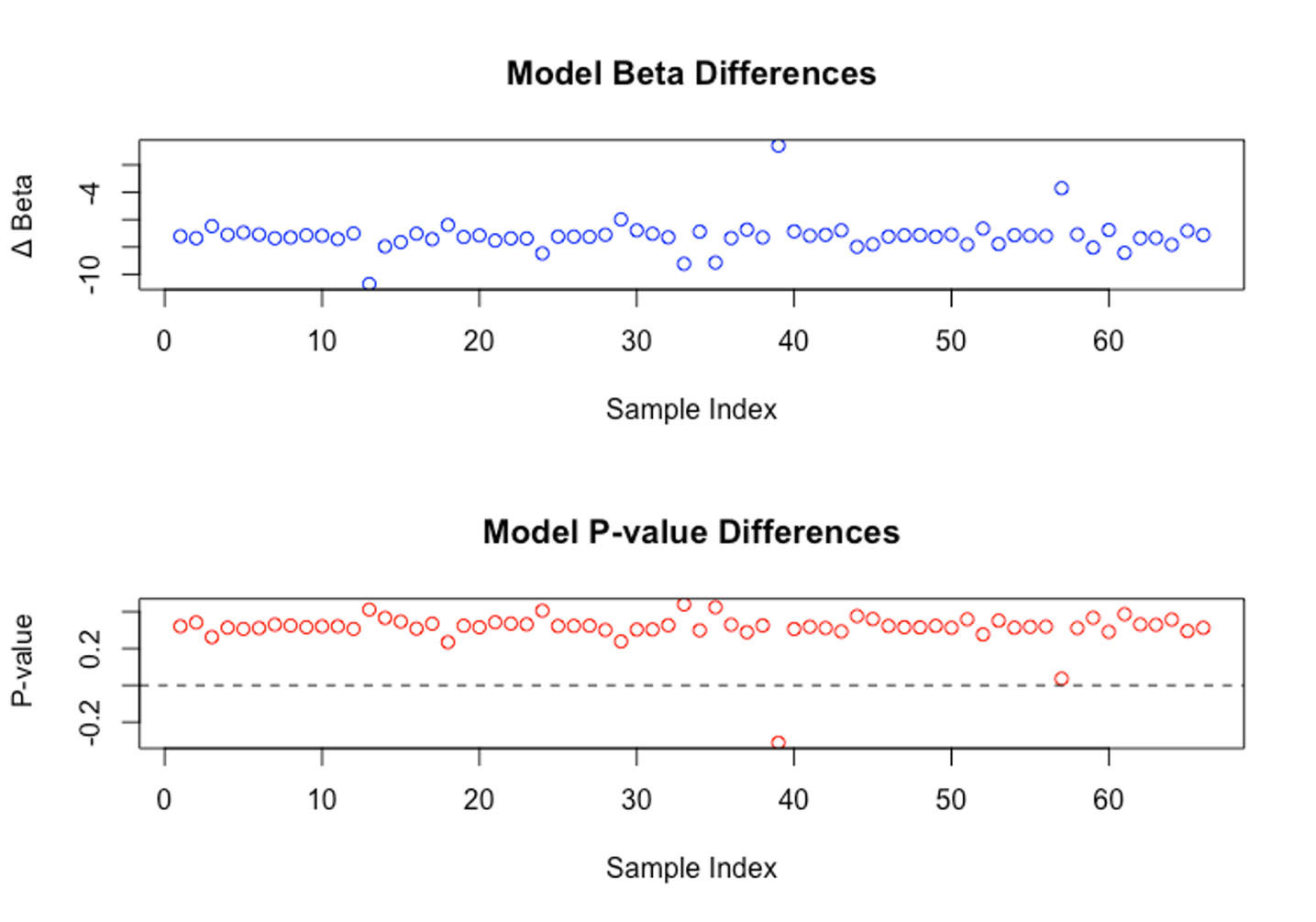


**Figure. A1.** Sensitivity Analysis of Linear Regression Model
